# The National NeuroAIDS Tissue Consortium Brain Gene Array: Two Types of HIV-Associated Neurocognitive Impairment

**DOI:** 10.1371/journal.pone.0046178

**Published:** 2012-09-26

**Authors:** Benjamin B. Gelman, Tiansheng Chen, Joshua G. Lisinicchia, Vicki M. Soukup, J. Russ Carmical, Jonathan M. Starkey, Eliezer Masliah, Deborah L. Commins, Dianne Brandt, Igor Grant, Elyse J. Singer, Andrew J. Levine, Jeremy Miller, Jessica M. Winkler, Howard S. Fox, Bruce A. Luxon, Susan Morgello for the National NeuroAIDS Tissue Consortium

**Affiliations:** 1 Department of Pathology, University of Texas Medical Branch, Galveston, Texas, United States of America; 2 Department of Neurology, University of Texas Medical Branch, Galveston, Texas, United States of America; 3 Department of Biochemistry and Molecular Biology, University of Texas Medical Branch, Galveston, Texas, United States of America; 4 Department of Internal Medicine, University of Texas Medical Branch, Galveston, Texas, United States of America; 5 Department of Neurosciences, University of California San Diego, La Jolla, California, United States of America; 6 Department of Pathology, University of Southern California, Los Angeles, California, United States of America; 7 EMMES Corporation, Bethesda, Maryland, United States of America; 8 Department of Psychiatry, University of California San Diego, La Jolla, California, United States of America; 9 Department of Neurology, University of California Los Angeles, Los Angeles, California, United States of America; 10 Department of Biostatistics, University of California Los Angeles, Los Angeles, California, United States of America; 11 Department of Pharmacology and Experimental Neuroscience, University of Nebraska Medical Center, Omaha, Nebraska, United States of America; 12 Department of Neurology, Mount Sinai Medical Center, New York, New York, United States of America; Institut National de la Santé et de la Recherche Médicale, France

## Abstract

**Background:**

The National NeuroAIDS Tissue Consortium (NNTC) performed a brain gene expression array to elucidate pathophysiologies of Human Immunodeficiency Virus type 1 (HIV-1)-associated neurocognitive disorders.

**Methods:**

Twenty-four human subjects in four groups were examined A) Uninfected controls; B) HIV-1 infected subjects with no substantial neurocognitive impairment (NCI); C) Infected with substantial NCI without HIV encephalitis (HIVE); D) Infected with substantial NCI and HIVE. RNA from neocortex, white matter, and neostriatum was processed with the Affymetrix® array platform.

**Results:**

With HIVE the HIV-1 RNA load in brain tissue was three log_10_ units higher than other groups and over 1,900 gene probes were regulated. Interferon response genes (IFRGs), antigen presentation, complement components and *CD163* antigen were strongly upregulated. In frontal neocortex downregulated neuronal pathways strongly dominated in HIVE, including GABA receptors, glutamate signaling, synaptic potentiation, axon guidance, clathrin-mediated endocytosis and 14-3-3 protein. Expression was completely different in neuropsychologically impaired subjects without HIVE. They had low brain HIV-1 loads, weak brain immune responses, lacked neuronally expressed changes in neocortex and exhibited upregulation of endothelial cell type transcripts. HIV-1-infected subjects with normal neuropsychological test results had upregulation of neuronal transcripts involved in synaptic transmission of neostriatal circuits.

**Interpretation:**

Two patterns of brain gene expression suggest that more than one pathophysiological process occurs in HIV-1-associated neurocognitive impairment. Expression in HIVE suggests that lowering brain HIV-1 replication might improve NCI, whereas NCI without HIVE may not respond in kind; array results suggest that modulation of transvascular signaling is a potentially promising approach. Striking brain regional differences highlighted the likely importance of circuit level disturbances in HIV/AIDS. In subjects without impairment regulation of genes that drive neostriatal synaptic plasticity reflects adaptation. The array provides an infusion of public resources including brain samples, clinicopathological data and correlative gene expression data for further exploration (http://www.nntc.org/gene-array-project).

## Introduction

The worldwide pandemic of Human Immunodeficiency Virus type 1 (HIV-1) infection began in the early 1980s. Morbidity and mortality often were due to central nervous system (CNS) pathology caused by CNS HIV-1 infection and opportunistic infections. Neurocognitive impairment (NCI) occurred most often in people with end-stage Acquired Immunodeficiency Syndrome (AIDS). The most severe form of neurocognitive impairment became recognized as the AIDS dementia complex, and later as HIV-associated dementia (HAD). HAD was associated strongly with active HIV-1 replication in the CNS and a neuropathological inflammatory response known as HIV encephalitis (HIVE) [1]. Strategies to pursue the pathophysiology and treatment of HAD focused on HIVE primarily, but up to half of the people with HAD do not exhibit HIVE at autopsy. Conversely, decedents with HIVE often were not demented [2], [3], [4]. The incidence of progression to end-stage AIDS and severe dementia was reduced after highly active antiretroviral therapy (HAART) was introduced. The prevalence of HIVE in autopsy surveys did not decline as sharply because the population of decedents is skewed to those at the end-stages [5], [6], [7]. With improved survival mild forms of NCI such as asymptomatic neurocognitive impairment and mild neurocognitive disorder remain prevalent while the prevalence of HIV associated dementia has declined [8], [9], [10]. In recent times up to half of subjects in well-studied populations treated with HAART still exhibit milder impairments, which was nosologically renamed HIV-associated neurocognitive disorders (HAND) in 2007 [11]. No more than 10% of HAART-treated autopsy cohorts - and probably much fewer in healthier cohorts - exhibit neuropathological evidence of HIVE [5], [6], [7]. Because HAND is substantially more prevalent than HIVE (e.g. 50% HAND versus less than 10% HIVE), the pathophysiology of HAND in most subjects needs to be re-evaluated [5].

To sort out overlapping pathophysiologies in HAND and HIVE, genome-wide screening of transcription regulation in brain tissue is a potentially powerful approach. Previous genome-wide surveys identified many abnormal gene transcripts in frontal neocortex of subjects with HIVE [12], [13], [14], [15], [16]. Key changes included upregulation of interferon response gene (IFRG) transcripts and innate immunity [12], [14], altered expression of microglial cell *CD163* [15], macrophage osteopontin [16], [17], neocortical presynaptic proteins [14], neocortical dopamine receptors and ion channels [13], [14], [18] and lysosome expansion with altered autophagy [16], [19], [20]. Due to limited access to clinically characterized decedents, prior study was limited to people with HIVE and a single brain sector (neocortex). The decline in the prevalence of HIVE, combined with an increased prevalence of HAND, means that brain gene expression patterns in most people with HAND, and in other brain sectors and circuits remain to be elucidated. To accomplish that a gene expression array was performed using clinically characterized brain specimens from the National NeuroAIDS Tissue Consortium (NNTC) [21]. The array was designed to provide a brain gene expression resource linked to publicly available brain specimens that were characterized neuropsychologically. Key questions were posed that could be addressed using NNTC resources: 1) How do the molecular changes in people with neurocognitive impairment and HIVE differ from impaired people without HIVE? 2) Is selective vulnerability of different brain regions and neural circuits important for gene expression, including differences in gray and white matter [20], [22], [23], [24], [25], [26]? 3) Are there any brain molecular changes in people who resist acquiring HIV-1-associated neurocognitive impairment?

## Results

### HIV Loads and Human Brain RNA Isolates

Three HIV-1 infected (HIV+) groups of subjects were compared to normal HIV negative (HIV-) controls (Table 1). HIV-1 RNA was detected in the brains of all three groups of HIV infected people (Groups B, C and D). Subjects with NCI and HIVE (Group D) had brain HIV RNA loads averaging over three log_10_ units higher than those without HIVE (Groups B and C) similar to another report [27]. (Note: The nosological diagnosis of HAND [11] was not yet formalized when group selection was made and patient testing was done. To avoid confusion regarding the changing nomenclature, we use “neurocognitive impairment NCI” to describe our subjects; HAND is reserved for the general discussion). Consistent with previous analyses of human brain tissue postmortem degradation of RNA did not exert a substantial influence on Affymetrix® gene array results [28], [29], [30]. Four post-hybridization quality checking parameters showed that brain mRNA was reasonably intact for this type of study (see Tables S1, S2, S3 and http://www.nntc.org/gene-array-project/quality-control). Preliminary calculations using Partek Genomics Suite were done examining the effects of excluding samples with degradation. These trials did not reveal any substantial influence overall. Accordingly, data from all 72 gene chips were included in the calculations presented. The raw Affymetrix data files have been deposited in the Gene Expression Omnibus repository (GSE35864).

**Table 1:** Subjects in the brain gene array.

Characteristic	Group Assignment			
	GROUP A	GROUP B	GROUP C	GROUP D
HIV-1 infection	HIV negative	HIV infected	HIV infected	HIV infected
Neurocognitive impairment	Not impaired	None or slight	Impaired	Impaired
Global impairment score*	Not applicable	4.0±1.4	6.9±1.1**	7.5±2.2**
HIV encephalitis (HIVE)	No HIVE	No HIVE	No HIVE	With HIVE
Brain HIV-1 RNA (log_10_ copies/g)				
White matter, frontal	0	3.53±0.69	3.85±2.54	7.40±1.80**
Neostriatum	0	3.84±0.50	3.51±1.37	7.27±0.84**
Neocortex, frontal	0	3.65±0.74	3.63±1.12	6.16±1.34**
Geographical breakdown***	NYC = 3	NYC = 1	NYC = 1	NYC = 1
	TEX = 2	TEX = 2	TEX = 2	TEX = 2
	UCSD = 1	UCSD = 2	UCSD = 2	UCSD = 2
	UCLA = 0	UCLA = 1	UCLA = 2	UCLA = 0
Age at death (years ± SD)	50.0±10.4	49.5±8.4	43.7±9.8	42.8±8.9
Race or ethnicity****	WT = 5; BL = 0HP = 1	WT = 6; BL = 0HP = 0	WT = 3; BL = 1HP = 2; MAS = 1	WT = 4; BL = 0HP = 1
Gender (M = male; F = female)	M = 5; F = 1	M = 6; F = 0	M = 7; F = 0	M = 5; F = 0
Brain freeze time (hours, median)	20.3	11.5	6.0	10.0
NNTC subject ID*****	A1, A2, A3A4, A5, A6	B1, B2, B3B4, B5, B6	C1, C2, C3, C4C5, C6, C7	D1, D2, D3D4, D5

* The Global impairment score is an index of overall performance on the NNTC neuropsychological battery of tests on the last assessment prior to death. A score ≤4 is considered normal; scores of 5 through 9 indicate progressively severe neurocognitive impairment. See Methods for details. Values are Mean ± SD.

** Mean ± SD; different from Group B using Student’s t test, p<0.00001.

*** Geographical locations are in the United States of America: NYC = New York City; TEX = Galveston/Houston, Texas; UCSD = San Diego, California; UCLA = Los Angeles, California.

**** WT = Caucasian; BL = African American; HP = Hispanic; MAS = Mixed Asian/WT.

***** The subject identifying codes shown here were assigned specifically for this study and match those given on the Gene Array page of the NNTC website (http://www.nntc.org/gene-array-project). Other identifiers also are assigned in NNTC archives. Samples of the brain specimens from these subjects are available to the public through the NNTC, and may have alternative identification codes.

### Variation According to Patient Group and Brain Region

**Figure 1:**
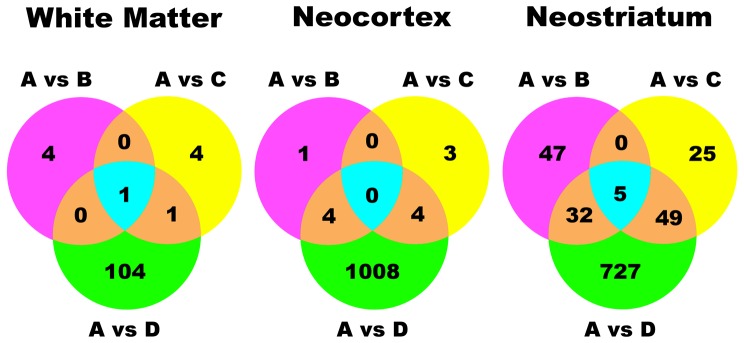
Venn diagrams of regulated probes on the array. The number of significantly regulated transcripts is shown in the three HIV-1 infected groups (Groups B, C, and D) as compared to uninfected controls (Group A) in three brain sectors. The criteria for significance employed a cutoff filter of ±2 fold change and p≤0.05 with the Benjamini-Hochberg correction for multiple testing comparisons using S+ArrayAnalyzer. Outer lobes represent probes regulated exclusively in one group. Regions within points of intersection represent probes regulated in more than one group. Group D (in green) contains NCI subjects with HIVE and had the most regulated genes in every brain sector. In neostriatum 91% of the probes with HIVE were upregulated. In neocortex 84% of the probes with HIVE were downregulated. Group C (yellow) contains NCI subjects without HIVE. Group B (purple) contains HIV-1-infected subjects without any NCI or HIVE. Groups B and C had few regulated probes that were delimited to neostriatum primarily. See Table 2 for the lists of regulated canonical pathways.

Multivariate modeling indicated that within the three brain regions the gene expression patterns could cluster the cases within each group (Figure S1). The potentially regulated transcripts in the 3 HIV-1 infected groups (B, C, and D) and three brain regions were tallied using S+ArrayAnalyzer software with a cutoff filter of ±2 fold and p≤0.05 with a Benjamini-Hochberg correction for multiple testing comparisons. Venn diagrams illustrate patterns of overlap in the HIV-1 infected groups in three brain regions (Figure 1). The subjects with NCI and HIVE (Group D) had over 1,900 potentially regulated transcripts in all brain sectors combined. In neostriatum upregulated transcripts were dominant with HIVE (745 out of 813). In frontal neocortex downregulated transcripts predominated strongly with HIVE (860 out of 1,016). In white matter there were comparatively few changes and upregulation predominated with HIVE (103 out of 118). Both of the HIV-1 infected groups without HIVE (Groups B and C) had less than 100 regulated probes each in all the sectors combined.

**Table 2:** Canonical Pathways regulated in regions of HIV-1 infected brain tissue.

Canonical Pathway*	Brain region					
	Neostriatum		Neocortex		White Matter	
	P value	Ratio	P Value	Ratio	P Value	Ratio
**CNS impairment with HIVE (Group A vs D)** *						
Interferon signaling ↑	<0.0001	0.41	0.0708	0.17	<0.0001	0.24
Activation of IRF cytosolic pattern recogn. receptor ↑	0.0030	0.14	0.0708	0.11	0.0054	0.05
Antigen presentation↑	<0.0001	0.41	- NS -		<0.0001	0.23
Pattern recognition receptors for bacteria & viruses ↑	0.0013	0.14	- NS -		0.0072	0.05
Complement system ↑	<0.0001	0.31	- NS -		- NS -	
Acute phase response signaling ↑	0.0037	0.10	- NS -		- NS -	
Hepatic fibrosis/hepatic stellate cell activation ↑	0.0050	0.10	- NS -		- NS -	
Toll-like receptor signaling ↑	0.0331	0.13	- NS -		- NS -	
GABA receptor signaling ↓	- NS -		0.0007	0.20	- NS -	
Clathrin mediated endocytosis ↓	- NS -		0.0045	0.11	- NS -	
Glutamate receptor signaling ↓	- NS -		0.0065	0.15	- NS -	
14-3-3 mediated signaling ↓	- NS -		0.0065	0.11	- NS -	
Synaptic Long term potentiation ↓	- NS -		0.0071	0.12	- NS -	
Cyclic AMP-mediated signaling ↓	- NS -		0.0129	0.11	- NS -	
Calcium signaling ↓	- NS -		0.0129	0.09	- NS -	
Axonal Guidance signaling ↓	- NS -		0.0141	0.08	- NS -	
Chemokine signaling ↓	0.1611	0.09	0.0214	0.13	- NS -	
Ephrin receptor signaling↓	0.0575	0.07	0.0224	0.09	- NS -	
Oxidative phosphorylation ↓	- NS -		0.0229	0.09	- NS -	
Protein ubiquitination pathway↑	- NS -		- NS -		0.0054	0.03
Caveolar mediated endocytosis ↑	- NS -		- NS -		0.0072	0.05
Liver X receptor/retinoid X receptor activation ↑	- NS -		- NS -		0.0417	0.04
**CNS impairment without HIVE (Group A vs C)** *						
None						
**No impairment and no HIVE (Group A vs B)**						
G-protein coupled receptor signaling ↓	0.0214	0.03	- NS -		- NS -	
Cyclic AMP-mediated signaling ↓	0.0288	0.03	- NS -		- NS -	

* Performed using Ingenuity software using probes that were changed more than two-fold and significant at p<0.05 corrected using Benjamini-Hochberg correction for multiple comparisons. NS, not significant. Ratio indicates the proportion of probes in the pathway that were significantly regulated. Up arrows denote increased expression. Down arrows denote decrease. Impairment with HIVE (Group D) is often referred to as Type I in the text and Table 3. Impairment without HIVE (Group C) is often denoted Type II.

### HIVE (Group D): Broad-based Immunological Activation

The list of significantly regulated canonical pathways using Ingenuity® Systems Pathways Analysis (IPA) is given in Table 2. The comprehensive list of significantly regulated probes appears in Supplemental material in a format suitable for data downloading (Table S4). Text mining with IPA showed that interferon response genes (IFRGs) and the interferon regulatory factor (IRF) pattern recognition canonical signaling pathways were upregulated in every brain sector (Table 2). Other strongly upregulated neuroimmune canonical pathways in IPA were antigen presentation and pattern recognition receptors for bacteria and viruses (in two brain sectors). Complement, acute phase inflammatory responses and toll receptor signaling pathways were strongly upregulated primarily in neostriatum. These upregulated pathways pertain generally to increased inflammation and neuroimmunity in HIVE. With HIVE there were 132 probes with at least a two-fold change in more than one sector (see Table S5). Most of these broadly regulated pathways in HIVE reflect pathological inflammation and increased neuroimmunity. Strong examples included ubiquitin-like modifier (*ISG15*); guanylate binding protein 1 interferon-inducible (*GBP1*); interferon-induced protein with tetratricopeptide repeats 3 (*IFIT3*), interferon-induced protein 44-like (*IFI44L*); butyrophilin subfamily 3, member A3 (*BTN3A3*); interferon-induced protein with tetratricopeptide repeats 3 (*IFIT3*), signal transducer and activator of transcription 1 (*STAT1*); myxovirus resistance 1, interferon-inducible protein (*MX1*); interferon induced with helicase C domain 1 (*IFIH1*); proteasome subunit, beta 8 (*PSMB8*); major histocompatibility complex, class I, B (*HLA-B*) (Table S5). All of these examples are interferon response genes (IFRGs) [31].

**Table 3:** Summary characteristics of proposed Types I and II HIV-associated neurocognitive impairment*.

Characteristic	Type I impairment	Type II impairment
NNTC Gene Array Group**	Group D	Group C
Last Global Impairment Score***	7.5±2.2	6.9±1.1
Neuropathological substrate	HIV encephalitis	Unknown
Related to brain HIV-1 replication	Yes	No
Influenced by HAART****	Decreased prevalence	Possibly no influence
Brain immune responses on array	Strong upregulation	Not upregulated, possibly deficient
Brain CD163 expression on array	Strong increase	Usually no increase
Neuronal mRNAs in neocortex on array	Broad downregulation	Not downregulated
Prevalence before 1995	At least 20%	Unknown
Prevalence 2011	Less than 10%	At least 35%

* IV, Human immunodeficiency virus

** NNTC, National NeuroAIDS Tissue Network

*** A Global Impairment Score ≤4 is considered normal. Scores of 5 to 9 represent progressively severe impairment. Mean ± SD

**** HAART, highly active antiretroviral therapy

**Figure 2:**
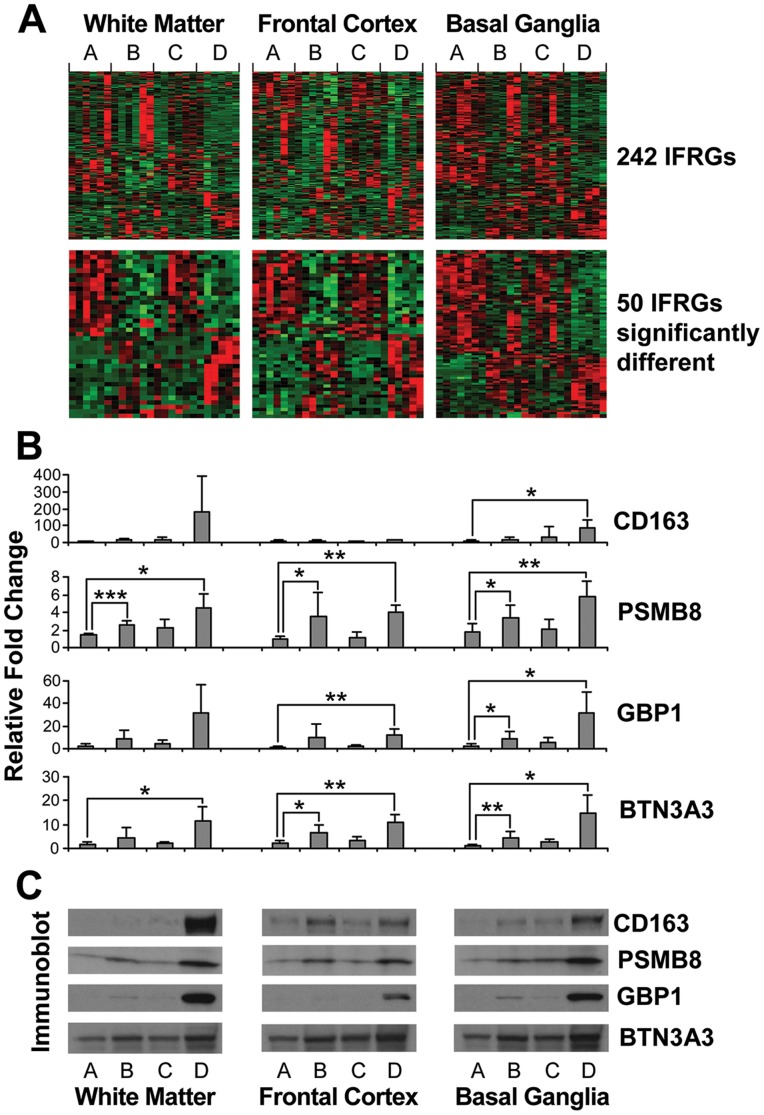
Interferon response genes (IFRGs) on the array. Four array groups are designated A, B C and D on the abscissa. The top panels contain heat maps of normalized signal intensities for 242 unselected IFRG probes, and for 50 IFRG significantly regulated probes. The heat maps illustrate clustering of IFRGs in the four groups. The B panels are histograms of real time PCR confirmation for three IFRG transcripts and *CD163* (mean ± SD, Student’s t test). The C panels illustrate confirmation at the protein level in Western blots of pooled extracts. Group D (NCI with HIVE) had significant regulation of IFRGs in all three brain sectors. Group C (NCI without HIVE) did not have significant IFRG regulation. Group B (HIV-1-infected subjects without any impairment or HIVE) had mild IFRG regulation. *p<0.05; **<0.01; ***<0.001.

Since IFRG and IRF pathway expression was robustly regulated a heat map was produced from normalized signals of established IFRGs in the literature [31] (Figure 2A). Subjects with HIVE (Group D) had sharply increased IFRG regulation. Subjects with neurocognitive impairment but no HIVE (Group C) lacked IFRG regulation. Subjects who were neuropathologically and neurocognitively normal (Group B) had a modest increase in some IFRGs that was significant when individual transcripts were confirmed using RT-PCR. Increased expression of three exemplary IFRG transcripts (*GBP1*, *PSMB8*, *BTN3A3*) is shown with comparison to *CD163*, which is a marker of activated brain microglial cells in HIVE [15] (Figure 2B). Confirmation at the level of protein expression in calibrated Western blots is given (Figure 2C).

### HIVE (Group D): Downregulated Neuronal Systems Only in Neocortex

**Figure 3:**
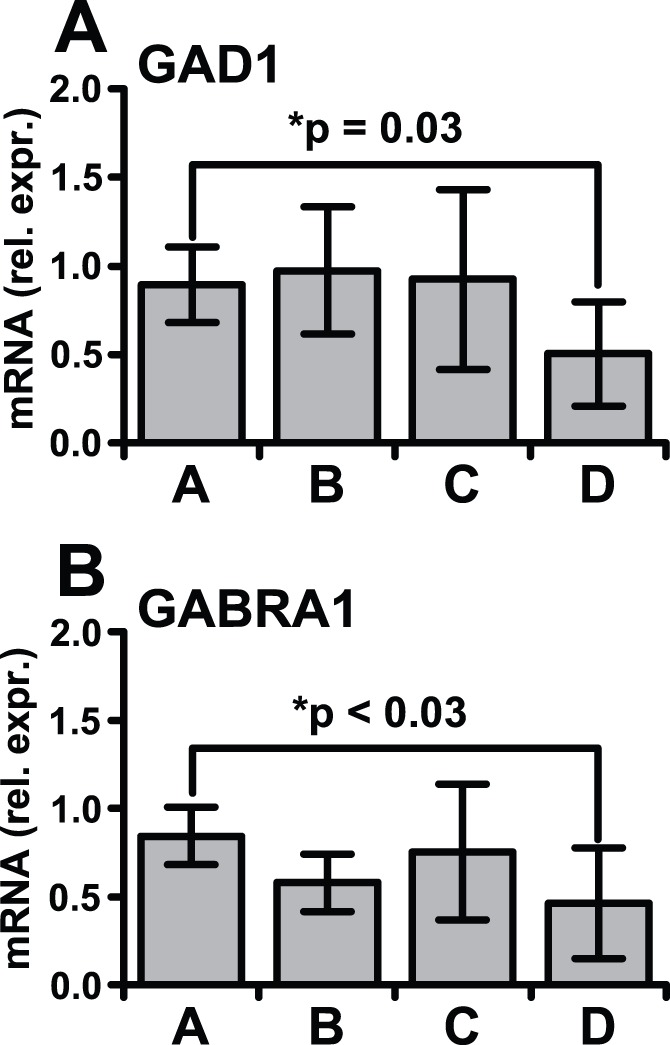
Confirmation of downregulated neocortical GABA pathway mRNAs in HIVE. *GAD1* codes for the rate limiting enzyme in GABA synthesis in presynaptic neurons (glutamic acid decarboxylase, 67 kDa). *GABRA1* codes for GABA receptor subunit A1 in postsynaptic neurons. Both of these mRNAs pertain to inhibitory neocortical neurons and were significantly decreased in frontal neocortex in subjects with NCI and HIV encephalitis (Group D). NCI subjects without HIVE did not have altered GABA transcripts (Group C). mRNA is expressed relative to GAPDH mRNA. Mean ± SD, Student’s t test.

Results from frontal neocortex with HIVE differed strikingly from neostriatum. Upregulation of IFRGs was significant in neocortex but the fold changes were weaker than other brain sectors (Table 2, Table S5). Instead, downregulated transcripts predominated by about 4 to 1 in frontal neocortex (Table 2, Table S4). The downregulated pathways are characteristically expressed by neocortical neurons. Some were recognized or implied in prior gene arrays using HIVE frontal neocortex, including synaptic receptors for GABAergic transmission, long term synaptic potentiation, glutamatergic neural transmission and chemokine systems [12], [14], [18]. Additional novel neuronal pathways were regulated in neocortex including clathrin mediated endocytic pathways, ephrin receptors, axon guidance, 14-3-3 and cyclic-AMP-mediated signaling pathways. GABA pathway transcripts were regulated the most. This was confirmed with RT-PCR using presynaptic (*GAD1*) and postsynaptic (*GABRA1*) GABA marker genes (Figure 3).

### Neurocognitive Impairment without HIVE (Group C)

**Figure 4:**
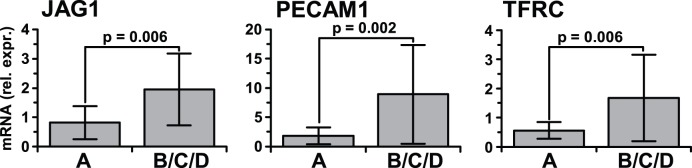
Increased endothelial cell type transcripts on the array. *JAG1*, *PECAM1* and *TFRC* are gene symbols for mRNAs that are expressed by brain endothelial cells predominantly. All three were significantly increased in Group C on the array. Confirmation with RT-PCR is shown. A, uninfected subjects (n = 6); B/C/D, HIV-1 infected subjects (n = 18). The increased expression of these endothelial transcripts was found to be present in the HIV-1 infected groups generally. mRNA is expressed relative to GAPDH mRNA. Mean ± SD, Student’s t test.

Just 92 probes were regulated in Group C primarily in neostriatum (Figure 1 and Table S6). There was a general lack of inflammatory type gene expression including IFRGs (Table 2), which is consistent with the fact that Group C lacked pathological inflammatory infiltration or high brain HIV-1 replication (see Table 1). No canonical pathway was significantly regulated in Group C in any sector using IPA. Neuronal pathways were not downregulated in neocortex in Group C (Table 2). One highly significant gene network was generated using unsupervised IPA network analysis that contained upregulated transferrin receptor (p90, CD71, *TFRC*), Von Willebrand factor (*VWF*), ceruloplasmin (*CP*), an endothelial cell tyrosine kinase (*TIE1*), a transcription activator (*RBM3*), complement factor H (*CFH*) and phosphatase 2A (*PP2A*), with convergence around MAP kinase 1 (*MAPK1*) and transforming growth factor beta 1 (*TGFB1*) (Table S6). All these transcripts are expressed characteristically in brain microvascular endothelial cells (ECs) [32], [33], [34], [35], [36], [37], [38], [39], [40] or perivascular cells (PCs) [41]. RT-PCR for three of these EC marker transcripts confirmed that they were indeed increased in HIV-1 infected subjects (Figure 4). The trend towards specificity for Group C using IPA was not significant using parametric statistics due to power limitations.

### HIV-1 Infection in Subjects Who were Normal Neurocognitively and Neuropathologically (Group B)

Prior gene arrays did not address whether HIV-1 infection *per se* (without NCI or substantial neuropathology) can influence brain gene expression (Group B). There were 94 regulated probes in this group; they were present in neostriatum almost exclusively (Figure 1, Table S7). Neuronal type genes were downregulated significantly in canonical pathways for G protein receptors and cyclic adenosine monophosphate (cAMP) signaling (Table 2). These pathways transduce slow synaptic transmission in postsynaptic neostriatal medium spiny neurons [42], [43]; their regulation experimentally is associated with synaptic plasticity [44], [45].

### Correlation with Viral Load

**Figure 5:**
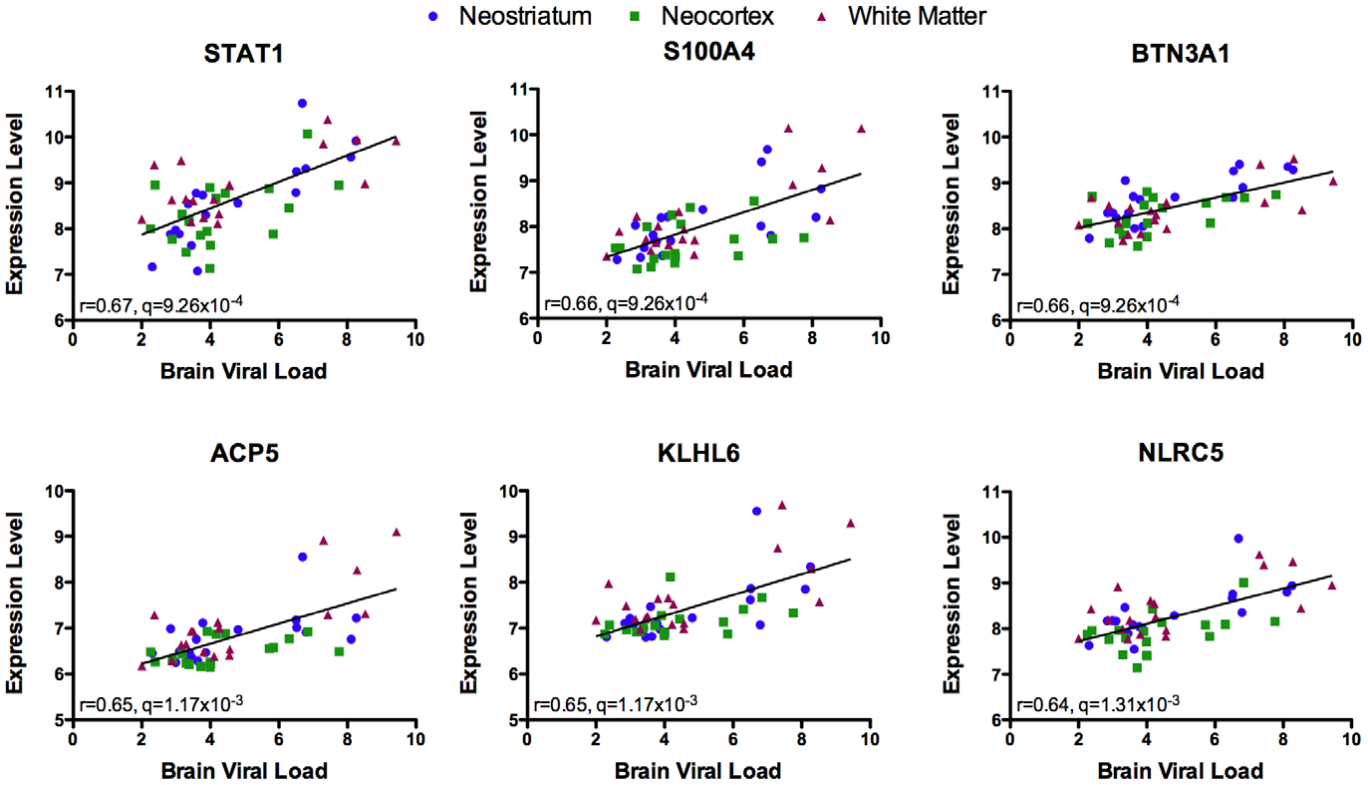
Correlation of gene expression with brain viral load. *STAT1*, *S100A4*, *BTN3A1*, *ACP5*, *KLJL6*, and *NLRC5* are gene symbols for the top six mRNAs that show significant correlation with brain viral load. The Pearson correlation coefficient (r) and p value are shown, along with a linear regression line modeling the correlation. The different brain regions are shown by different colored and shaped symbols.

As brain viral load is higher in HIVE (Group D) than in HIV infected groups without HIVE (Groups B and C) the relationship between HIV levels and gene expression in the brain was investigated. Correlation analysis was performed between viral load and gene expression in all samples from infected individuals. Over two thousand probe sets representing 1,960 genes showed a significant correlation with p<0.01 (Table S8, Figure 5). Of these there were 127 genes with a positive correlation of r>0.5, and 32 genes with a negative correlation of r<−0.5. The top canonical pathway identified by IPA for the genes correlated with viral load is interferon signaling (p = 3.72×10^−6^), consistent with its role in viral infection. Similarly related to infection are other enriched pathways for antigen presentation (p = 2.14×10^−3^) and activation of interferon regulatory factors by cytosolic pattern recognition receptors (p = 5.50×10^−3^).

### Candidate Biomarkers of Neurocognitive Impairment

Brain gene expression might reflect downstream events in a “final common pathway” of neuronal degeneration in subjects with NCI. This is a highly salient question because it would support to the notion that there is a common cliniconeuropathological sequence of neurodegeneration in HIV-infected people. Venn diagrams (Figure 1) illustrated that 54 individual neostriatal transcripts were regulated in the two groups with NCI (C and D). No significant regulated canonical pathways were identified using IPA. One significant gene network was identified by text mining with IPA in which immunoglobulin gene transcripts in B lymphocytes are upregulated via nuclear factor kappa B (NF-κβ) (i.e. *IGHV4-31*, *IGHM*, IGL@, *IGLJ3*) (Table S7). Systemic B cell activation and hypergammaglobulinemia in HIV/AIDS is well known [46]; a potential relationship to brain dysfunction has not been suggested heretofore.

## Discussion

This human brain gene array study benefited from the utilization of brain specimens from neurocognitively well-characterized persons, attention to the impact of HIV on neurocognitively normal individuals, inclusion of subjects without encephalitis, an emphasis on regional brain variation and the measurement of brain HIV-1 concentration. The results provide several new insights pertaining to cognition, neurovirology, neuroimmunology, and brain circuit dysfunction. These particular advantages should be weighed against some inherent limitations of this study. Having 12 different groups and/or brain sectors diluted the overall statistical power of the 72 chips and made it difficult to exclude the null hypothesis for borderline changes, especially in Groups B and C which had few regulated transcripts. As well, the strong emphasis on pathological outcomes and brain HIV loads meant that some longitudinal clinical details regarding HAART treatment, plasma CD4+ lymphocytes, and plasma HIV loads could not be obtained for comparison to array results. A further limitation is that cross sectional outcomes at autopsy are difficult to compare to neurocognitive function because both could fluctuate over time [8], [9], [10]. These aspects should be kept in mind when considering the cliniconeuropathological correlations discussed below.

A critical question posed was whether molecular changes in the brains of people with neurocognitive impairment without HIVE differ from impaired people with HIVE. The results provide compelling evidence that there are indeed two highly different patterns of brain gene expression in people with HIV-associated neurocognitive impairment. The differing molecular patterns suggest fundamentally different pathophysiologies in subjects with virtually identical neuropsychological phenotypes. Although the two patterns can only be recognized post-mortem at present, they imply that lumping together of clinical impairment phenotypes into one nosological entity (i.e. HAND) [11] may be problematic [5], [47]. A key caveat to that suggestion is that HIVE and inflammatory changes could have been present transiently in Group C but were not present at autopsy. If that were true, then HAND without HIVE could reflect permanent neuronal loss (neurodegeneration) that persisted after HIVE and HIV-1 replication were normalized using HAART. Some evidence to the contrary can be cited. First, two neuropathological outcomes were suggested long before the era of HAART when there was relentless clinical progression to end stage AIDS without interruption [2], [3], [4], [5]. Second, HAART era autopsies show that “burnt out HIVE” occurs [48], but the astroglial scars and other lasting neuropathological residua were not present at autopsy. Third, some commonality pertaining to neuronal transcripts is expected if both scenarios are the result of permanent neurodegeneration, but no pathway related to neurodegeneration was significantly regulated in Groups C and D both. It remains possible that markers of neuronal injury not measured in this study (e.g., injury to synapses and dendrites [49]) could underlie the NCI in both groups C and D. Nevertheless, brain gene expression lends substantial support to the proposal that two clinicopathological scenarios occur that we designate here as Type I NCI for those with HIVE, and Type II for those without HIVE (Table 3). Clinically both scenarios would be assigned an equivalent nosological diagnosis (HAND) using current nomenclature [11]. Our subjects underwent autopsy before the currently used clinical nosology (“HAND”) was adopted, but the implications of our results apply to HAND because the criteria for diagnosing neurocognitive impairment are generally equivalent.

Recognizing a formal cliniconeuropathological distinction between suggested types of HAND has translational implications because the types of therapeutic remedies and diagnostic approaches may be different. Decreasing HIV-1 replication in the brain with HAART type medicines is likely to be beneficial to prevent Type I impairment because it could decrease brain HIV-1 replication, decrease broad inflammatory responses as reflected by IFRG and IRF responses and provide neuroprotection from inflammatory stress. With Type II impairment reducing brain HIV-1 replication is less promising. The concentration of HIV-1 RNA in the brain of these subjects was not higher than unimpaired people (Group C versus Group B, see Table 1). Pursuing a mechanism to distinguish these two kinds of impairment in the clinic is a key research priority. Experimentally, postulating two pathophysiological categories of HAND has implications for interpreting the comparative pathology. Specifically, Simian Immunodeficiency Virus Encephalitis (SIVE) is an excellent model of HIVE that can be used to assess CNS responses to treatment in Type I impairment. Its ability to address Type II impairment is not as clear-cut [50], [51]. The same applies to *in vitro* cellular reconstitution models of HAND, which address neurovirological and neuroimmune changes [52] that are not observed at autopsy in Type II impairment. The potential limitations of encephalitic and *in vitro* models deserve heightened consideration.

### Type I Neurocognitive Impairment (Group D)

Neurocognitive impairment with HIVE is the best known cliniconeuropathological scenario. HIVE often is assumed to be the presumptive cause of HAND even though a very substantial proportion of subjects do not exhibit HIVE at autopsy [2], [3], [4], [5]. HIVE was characterized on the array by 1) broad-based upregulation of IFRGs, complement, antigen presentation and many other inflammatory response gene pathways, especially in neostriatum; 2) broad-based downregulation of neuronal transcripts selectively in frontal neocortex, including pre- and post-synaptic GABAergic inhibitory systems and other synaptic elements; 3) a high burden of replicating HIV-1 in all three brain sectors examined. Inflammatory cell infiltration and activation, virus replication and astroglial hypertrophy are present in HIVE and remain key concepts [1], [5], [47], [48], [52], [53]. To some extent, sharply increased inflammatory responses reflect changes in the cellular composition of a pathologically inflamed brain specimen. The upregulation of IFRGs, antigen presentation, the complement system, *CD163* antigen and other neuroimmune systems were noted on another human brain gene array that was limited to frontal neocortex [14] (see Figure S2 for a direct comparison). Activation of the interferon system has also been found in animal models of HAND [54], [55], [56], [57].

Hundreds of downregulated neuronal transcripts in neocortical neurons also were observed in type I impairment. The altered inhibitory GABAergic system was apparently specific to neocortical neurons and is reflected histologically as a loss of calbindin immunoreactive neurons in neocortex, but not neostriatum [58]. The selectivity of neocortical GABAergic neurons is notable because neostriatal neurons are predominantly GABAergic [59] yet were not regulated in step with the interneurons in frontal neocortex. Presynaptic and postsynaptic GABA markers both were regulated (Figure 3), which implies coordinated changes in synaptic tone (plasticity) in inhibitory neocortical circuits. Downregulation of neocortical GABAergic circuits is a recognized feature in many subjects with frontal lobe dysfunction phenotypically similar to HAND. Schizophrenia [59], [60], [61], [62], depression and anxiety [60], [63], [64] and substance abuse disorders [65] can produce a similar change. The lack of GABAergic inhibitory control leads to heightened excitatory output from frontal neocortex [59], [62], which agrees with some electroencephalographic recordings made in patients with HIV/AIDS [66]. Regulation of neocortical GABAergic circuits in HAND may, therefore, reflect a generic change when frontal lobe output is abnormal.

Regulation of novel canonical pathways in neocortical but not neostriatal neurons (Table 2) illustrates a striking selective vulnerability of neuronal populations in HIVE [67]. These perturbed pathways might be related to neuropathological changes in HIVE, including axonal integrity, synaptodendritic pathology, and synaptic potentiation, which all are relevant to HIVE [1], [49], [68], [69]. The spatial disconnection between innate immune responses (weak in neocortex and strong in neostriatum) and neuronal gene regulation (stronger in neocortex and weak in neostriatum) runs contrary to the typical paracrine model of HIV dementia, in which immune cells and neurons are reconstituted *in vitro* in close proximity to each other [52], [53]. Increased focus is needed on neocortical circuit level changes in subjects with Type I impairment and HIVE.

### Type II Neurocognitive Impairment (Group C)

The pathophysiology of neurocognitive impairment without HIVE remains virtually unknown. This is a critical gap because Type II impairment is most prevalent today [8], [9], [10] although it was well documented prior to the era of HAART [1], [2], [3], [4], [5]. Brain gene expression in Type II impairment was characterized by low brain HIV burden, virtually no evidence of increased IFRG responses, no downregulation of transcripts in neocortical neurons, and a relative paucity of regulated transcripts overall (Table S6). Just one pattern of brain gene expression was recognized in this group, with transcripts characteristically expressed by vascular and perivascular-type cells being regulated in neostriatum. Brain microvascular ECs are exposed directly to blood plasma and are potentially vulnerable to systemic and metabolic anomalies including inflammation [70], [71]. Accordingly, many of the regulated EC transcripts in Group C undergo changes in response to vascular stress or injury. Soluble EC markers in blood plasma are increased in HIV/AIDS, which lends support to a suggested role for systemic EC dysfunction [72], [73], [74], [75]. ECs conduct transvascular signaling from the plasma compartment to brain cells such as neurons and astrocytes via the neurovascular unit, often in response to infection or inflammatory and metabolic events [70], [71], [76]. As well, transvascular modulation leads to leakage of blood plasma through the blood-brain barrier (BBB) in HIV/AIDS, which is correlated significantly with HAD [77], [78], [79]. Plasma components that drive transvascular changes and are linked with HAND include plasma lipopolysaccharide (LPS) released by gut bacteria and soluble CD14 antigen [80]. The suggested role of LPS in HAND is supported experimentally by studies which show that plasma LPS drives many brain immune responses and EC changes [81], [82], [83]. All told the array results indicate that EC changes in the brain point to neurovascular stress in HIV/AIDS, very often without encephalitis or obvious neuropathological changes. These changes could be useful clinically to investigate and monitor the implied microvascular roles in Type II impairment, or to distinguish Type I from Type II impairment clinically.

### Brain Gene Expression and No CNS Impairment (Group B)

An important question addressed pertains to brain genes expressed in people who resisted becoming impaired to the end stage of AIDS (Group B). Such genes could be reflective of innate or acquired factors that confer resistance to HAND. Mild changes in neuroimmunological transcripts in Group B were similar in type and anatomical distribution to HIVE (Figure 2, Table S7). The meaning of these lower-level inflammatory changes without CNS impairment remains unclear because HIV-1 replication in the brain was not increased in Group B (see Table 1). Canonical pathways uniquely regulated in Group B were limited to the neostriatum. Regulation of neostriatal transcripts suggests that striatal circuits were perturbed when neuropsychological impairment was not clinically apparent. In turn that implies that neostriatal circuits are highly plastic in HIV/AIDS and confer circuit level accommodation and apparent “resistance” to impairment. Modification of dopaminergic neostriatal circuits in HIV/AIDS may be a beneficial adaptation (versus a deleterious mechanism of dysfunction) [13], [84], [85]. That suggestion has garnered some support from functional brain imaging studies, in which task-specific adaptive changes in frontostriatal circuits occurred without any morphological or neuropsychological changes [86], [87]. The array results provide novel glimpses into some neostriatal synaptic systems that could be involved with successful accommodation. These potentially beneficial changes in neural circuitry could be emulated pharmacodynamically, especially in vulnerable subjects.

### Brain Gene Expression in White Matter

Imaging surveys indicate a very high prevalence of white matter changes in HIV-1 infected cohorts [22], [24]. White matter pathology can be especially severe in HIVE, and its pathophysiology is of particular interest [20], [22], [23], [24], [25], [26]. We find that the strongest regulated pathway in white matter was Class I histocompatibility antigen presentation in Type I impairment.

While this manuscript was under review a study of gene expression in the white matter of subjects from the NNTC was published [88]. Four of the subjects were in common with our study (two uninfected controls: our A1 and A3 are their C1 and C3, one impaired non-HIVE: our C1 their HAND6, one impaired HIVE: our D1 their ART2). In this other study gene expression profiles of impaired subjects who were on antiretroviral treatment at the time of death differed from those that were not, regardless of the presence of HIVE [88]. Similar to our findings in Group D, increased inflammatory (antiviral and immune response) pathways were present in those dying off treatment, as well as decreased synaptic transmission and neurogenesis, the former of which we found in neocortical samples but not white matter (Table 2). In subjects dying on treatment many fewer dysregulated genes were found, but these still included genes involved in the immune response, including interferon pathways [88].

In our subjects we did not have information on use of treatment at death, but rather whether or not they had been treated with antiretrovirals. Only two subjects (in Group C, C2 and C4) had not been treated. While one of the subjects in common between the studies (C1 in our study, HAND 6 in the other) had an extensive history of treatment (14 different antiretroviral medications), this subject died off treatment [88]. Due to the lack of more complete information we were unable to examine this factor in our work.

### Correlation of Gene Expression and Viral Load

We found extensive correlations between the level of brain virus and genes, with substantial enrichment for those involved in the host response to viral infection (Table S8, Figure 5). Although recently published work [88] did not find gene expression correlations with brain viral load, it is important to note that our correlation study included neocortex and neostriatum in addition to white matter, increasing our power. Even so, examining the three regions separately still yielded significant correlations to viral load in each region including the white matter (Table S9). Clearly the characterization of molecular changes involved with CNS impairment in HIV infection will require further work examining the effect of brain region, virus, treatment, clinical parameters, as well as other co-morbid factors.

### Interface with NNTC Specimens and Data

A key feature of the NNTC brain gene array is that the brain specimens from the selected subjects are available to the public for confirmatory biochemical, molecular and histological study. In addition tissue from lymphoid and other organs are available from many cases for comparative studies. Raw array data are available from the NCBI Gene Expression Omnibus (GEO accession#GSE35864) for reanalysis and further exploration, and additional information on these cases and this study is available from the NNTC (http://www.nntc.org/gene-array-project).

## Materials and Methods

### Study Subjects and Ethics Statement

The cases for this study were selected from over 500 banked brain specimens in the National NeuroAIDS Tissue Consortium (NNTC) that had full antemortem neuromedical characterization [21]. NNTC studies were conducted in accordance with human subject protection protocols at participating institutions. Written consent was obtained for subjects at four collection sites in the USA. The following offices maintained the institutional review boards (IRBs) that provided oversight for the protection of human subjects: 1) The University of Texas Medical Branch Office of Research Subject Protections; 2) Mount Sinai Medical Center Program for the Protection of Human Subjects; 3) University of California, San Diego Human Research Protections Program; 4) University of California, Los Angeles Office of the Human Research Protection Program. These IRBs reviewed the protocol at regular intervals and gave written approvals continuously over 12 consecutive years to the present. As it was financially infeasible to perform a genome-wide survey on all cases, four groups with the following characteristics were examined (Table 1). Group A (N = 6): HIV-1 uninfected with no neuropathological abnormalities at autopsy; Group B (N = 6): HIV-1-infected (HIV+) neuropsychologically normal with no neuropathology; Group C (N = 7) HIV+ with substantial HIV-associated neurocognitive impairment (HAND) as defined below, with no encephalitis (HIVE) or substantial neuropathological defect; Group D (N = 5) HIV+ with HAND and HIVE. After its selection one of the HIVE cases was found have CNS co-infection with other viruses (Cytomegalovirus and Papovavirus). Analysis of the gene array results of the HIVE cases (Group D) using principal components analysis revealed that this case (D5) is similar to the other samples in the group (Figure S3), and thus it remained included in the analysis. In addition to the four cases noted in the text to overlap with the recently published array study [88], a sample from one additional case had been examined in another study array [14]. Neurocognitive assessments were performed prospectively within six months of death as per NNTC protocol [21]. The four NNTC sites used equivalent protocol to evaluate neuropsychological test performance and participated in a quality assurance program that minimized intersite variability [89]. Autopsies were performed using a uniform protocol established by the NNTC and the collection sites maintained a neuropathology quality assurance program [21]. The diagnosis of HIVE was made according to Budka [1]. Groups were matched as best as possible according to availability with respect to gender, age, ethnicity, postmortem times and pertinent variables as indicated (Table 1).

### Neuropsychological Assessment

All subjects received at least one comprehensive neurocognitive evaluation approximately 6 months before death. The assessment is fully described elsewhere [21], [89] and included tests of attention and working memory, speed of information processing, executive functions, memory, language and motor function. The test results were transformed to age-education-corrected standard scores (T scores) and then assigned a “deficit score” (DS) based on the deviation from expected in an unimpaired population as follows. A T score >39 (not more than one standard deviation below mean) was assigned a DS of 0, no impairments; T = 35−39 (DS = 1, mild impairment); T = 30−34 (DS = 2, mild to moderate impairment); T = 25−29 (DS = 3, moderate impairment); T = 20−24, (DS = 4, moderate to severe impairment; T<20 (DS = 5, severe impairment). These test results allowed for determining impairment in specific cognitive domains and facilitated a clinical rating system to categorize global cognitive impairment, as follows: A global impairment score (also known as global clinical rating or GCR) of 1–4 indicated performance ranging from above average to borderline normal; scores between 5 to 9 indicated progressively severe abnormality. For purposes of this study cases rated 1 to 4 were termed cognitively unimpaired; those rated 5 or 6 were mildly to moderately impaired; those rated 7 to 9 were severely impaired (see Table 1).

### Specimen Processing and RNA Extraction

Three sectors of the frozen brain specimens were evaluated. 1) Frontal neocortex at Brodmann Area 9; 2) neostriatum sampled at the head of the caudate nucleus; 3) white matter from deep frontal lobe. Approximately 0.5 g of frozen tissue was removed with a Dremel® tool with a porcelain blade or a disposable blade (Dremel, Tylertown, MS). One milliliter of Trizol® (Invitrogen, Carlsbad, CA) and 400 µl of silica microbeads (0.5 millimeter) was added and the sample was pulsed four times for 20 seconds in Microbeadbeater (Biospec, Bartlesville, OK, USA), with cooling on ice between pulses. 200 µl of chloroform were added at 25°C and the sample was centrifuged for 15 minutes at 12,000×g. Colorless supernatant was mixed with 700 µl of isopropyl alcohol for 10 minutes at 25°C and centrifuged at 4°C for 10 minutes at 12,000×g. The RNA pellet was washed in 1 milliliter of 75% ethanol, pelleted in the cold, dried in air and resuspended in 100 µl of DEPC water. The solution was digested at 25°C for one hour with DNase using Promega RNase free-DNase in a volume of 200 µl; the reaction was halted by adding 200 µl of phenol/chloroform (% v/v). The 12,000×g supernatant was mixed with an equal volume of isopropanol, frozen to −20°C for 20 minutes and centrifuged at 4°C for 15 minutes at 16,000×rpm. The RNA pellet was decanted, washed with 300 µl of 75% ethanol, dried in air for less than 5 minutes, resuspended in 100 µl of DEPC water and then stored at −80°C. RNA concentration and optical densities at 260 and 280 nm were determined using an Eppendorf Biophotometer (Hamburg Germany). Purification to remove small RNA fragments was done using a Sephadex G-25 Quick Spin column (Piscataway, NJ, USA). RNA was quantified with NanoDrop ND-1000 (NanoDrop Technologies, DE, USA). Purified RNA banding was assessed qualitatively with an Agilent BioAnalyzer 2100 (Agilent Technologies, CA, USA).

### RNA Hybridization

All 72 samples were subjected to gene expression analysis using Affymetrix “Gene Chips” (human 133 plus 2.0). Briefly, each gene is represented by a set of probe pairs (11–20 pairs per gene) composed of a 25 base oligonucleotide. Each probe pair contains one “perfect match” oligomer of the target mRNA, and one “mismatch” probe that differs at position 13, to control for non-specific hybridization. Ten to fifteen micrograms of total RNA were used as the template for first-strand cDNA synthesis using reverse transcriptase and an oligo dT primer for bacteriophage T7 RNA polymerase promoter. To convert cDNA to double stranded DNA templates second stand synthesis was done using T7 RNA polymerase and biotin-tagged nucleotide triphosphates (NTPs). For standardization, polyadenylated RNAs representing eight prokaryotic genes (dap, thr, lys and phe from *Bacillus subtilis*, bio B, bio C and bio D from *Escherichia coli* and cre from bacteriophage P1) were spiked to each total RNA sample before first strand cDNA synthesis. Spiked RNAs were added at different copy numbers in each RNA sample for standardization and to assess the effectiveness of labeling and hybridization of these probes on the array. Hybridization was performed using biotin-labeled cRNA at 45°C for 16 hours in 0.1 M MES, pH 6.6, 1.0 M NaCl, 0.02 M EDTA and 0.01% Tween 20. Gene expression was analyzed using the Affymetrix® GeneChip® Analysis software (GCOS). An “Absolute Analysis” for each chip was used to determine what genes were expressed in the total RNA sample. A comparison of the results from two separate gene chips was performed to determine differences in the level of expression of each gene between two samples (see www.scmm.utmb.edu/genomics/index.htm). Post-hybridization screening was performed to assess the condition of the mRNA and its hybridization properties [90]. Four patterns of hybridization were calculated for each chip: 1) Scaling Factor; 2) Background average; 3) Percent present; 4) 3′to5’ ratio for GAPDH. Details of these RNA quality checking parameters and complete results for 72 chips are provided in supplementary data (Tables S1, S2, S3). The data from the arrays have been deposited in GEO, accession # GSE35864.

### Brain RNA and Quantitative Polymerase Chain Reaction (qPCR)

Detection of the HIV-1 envelope protein mRNA in total RNA extracts from brain tissue was performed using a modification of a single copy procedure [91]. Brain RNA was extracted using RNeasy Lipid Tissue Mini Kit (Qiagen, Valencia, CA, USA). One µg of brain RNA and 1 µmol/L of anti-sense primer 84R were used in 20 µl reactions (iScript cDNA Synthesis Kit, Bio-Rad, Hercules, CA, USA). Four µl of cDNA was used for a 25 µl real-time PCR using JumpStart Taq ReadyMix (Sigma, Saint Louis, MO, USA) and SmartCycler (Cepheid, Sunnyvale, CA, USA). HIV RNA copies were computed using constructed standard curves against known brain secondary standard dilutions and expressed relative to the wet weight of the brain sample. The following host genes were analyzed with real time PCR: *CD163* (Hs01016661_m1), *GBP1* (guanylate binding protein 1, Hs00977005_m1), *PSMB8* (proteasome subunit beta 2i or LMP7, Hs00188149_m1), *BTN3A3* (butyrophilin 3A3, Hs00946540_gH), *GABRA1* (gamma-aminobutyric acid receptor subunit alpha-1, Hs00168058_m1), *GAD1* (glutamic acid decarboxylase, Hs01065893_m1), all using oligonucleotides primers and probe mix from Applied Biosystems, Foster City, CA, USA and real time PCR reagents as for HIV-1. The endothelial cell transcripts *JAG1* (Jagged 1), *PECAM1* (platelet endothelial cell adhesion molecule-1), and *TFRC* (transferrin receptor protein) were analyzed using SYBR Green JumpStart Taq ReadyMix for Quantitative PCR (Sigma, Cat. S4438, Sigma, Saint Louis, MO, USA). The primers for *JAG1* (TCCAAAAGGCTTATAAAACAGAGT and GCCAAGAACAACACATCAAAGA), *PECAM1* (CACAGATGAGAACCACGCCTA and TTTAATACAACATCCACGAGGGT), *TFRC* (CTAGAACTTGCATGACCTTTACTGT and AAGGAATCTCCACACTGTTGCA) were from Sigma Genosys (The Woodlands, TX, USA). One or 2 µl of cDNA was used for PCR. Results were normalized to *GAPDH* (GAPDHf, GAAGGTGAAGGTCGGAGTC; GAPDHr, GAAGATGGTGATGGGATTTC; GAPDHp, Fam-CAAGCTTCCCGTTCTCAGCC-TAMRA (Sigma Genosys). PCR conditions were 95°C, 15 seconds, 60°C, 60 seconds; 40 cycles. Fold changes were calculated using the ΔΔCt method.

### Semiquantitative Western Blotting

Protein was extracted from 0.2 grams of whole brain samples homogenized in 3 volumes of standard homogenization buffer (10 mM Tris-HCL pH 7.8, 0.5 mM DTT, 5 mM MgCl_2_ and 0.03% Triton X-100) containing protease inhibitor cocktail (Sigma-Aldrich, St. Louis, MO, USA) and phosphatase inhibitor cocktail set II (Calbiochem, San Diego, CA, USA). Homogenization was done with three 20 second pulses using zirconia/silica 0.5 mm microbeads in a Minibeadbeater (BioSpec Products, Bartlesville, OK, USA). Homogenates were mixed with an equal volume of 2× Laemmli Sample Buffer (Bio-Rad Laboratories, Hercules, CA, USA), boiled for 5 minutes and loaded on 4–20% gradient Tris-HCL polyacrylamide gel for SDS-PAGE. Immunoblotting was done using antibodies and dilutions as follows: CD163 at 1∶1000 (Novocastra, NCL-CD163, Newcastle upon Tyne, UK); Guanylate binding protein 1 (*GBP1*) at 1 ug/ml (MBL International, K0144-3, Woburn, MA, USA); LMP7 (*PSMB8*) at 1∶1000 (BIOMOL, PW8845, Plymouth Meeting, PA, USA); butyrophilin 3A3 (*BTN3A3*): at 1∶1000 (Sigma-Aldrich, HPA007904, St. Louis, MO, USA). Each gel run was calibrated with secondary standards. Densitometry was performed using OneD Scan (ScanAlytics, Rockville, MD, USA).

### Statistical Approaches

Partek Genomics Suite (Partek Incorporated, MO, USA) was used in the initial screening to compare uninfected pathologically normal brain specimens (Group A) to the other three groups. Three comparisons were calculated (A vs B, A vs C, and A vs D) for all three brain sectors for a total of nine operations. The ANOVA was calculated on normalized hybridization signals from.cel files. For the initial first low-stringency sorting, differentially expressed genes were filtered for p-values of ≤0.05 and a fold change of ±2 (log2 ratio between −1 and 1), without a correction for multiple comparisons. Partek was also used for multivariate modeling, using the partial least squares regression technique [92] for all four groups; to perform correlation with viral load (Groups B, C and D) calculating Pearson’s correlation coefficient (r) and p value between the viral load in each specimen (log_10_) and the corresponding probe set expression values (log_2_); and the principal component analysis for the cases in group D.

Calculations for the final differential expression analysis were performed using S+ArrayAnalyzer software (TIBCO, Palo Alto, CA, USA), using rma background correction method and quantiles normalization. Local-pooled-error tests were used to determine significance [93]. The false discovery rate was set at 0.05 according to the method of Benjamini and Hochberg [94]. Probes were considered significant if the fold change was ±2 and the false discovery rates was less than 0.05 relative to normal brain tissue (Group A). Significant regulation of canonical pathways and regulated networks of gene interactions were identified using the IPA Knowledge Base (Ingenuity Systems, Redwood City, CA, USA) [95]. Network discovery analysis was undertaken and interaction-based relationships in the IPA Knowledge Base were constructed. Venn diagrams of regulated probes were constructed using a parsing program written using PERL. Heat maps were constructed from normalized signals using Spotfire® DecisionSite (TIBCO).

## Supporting Information

Figure S1 **Partial least squares regression analysis.** Global gene expression in the four groups (A: HIV negative; B: HIV positive without NCI or HIVE, C: HIV positive with NCI without HIVE, D; HIV positive with NCI and with HIVE) was analyzed within each brain region. Graphs show the distribution of samples in respect to the top three principal components.
(TIF)

Figure S2 **Neocortical gene expression in this study compared to prior array results**. Expression in the array is similar to the array of Masliah et al (reference 14). Both studies compared HIV infected brain specimens without HIVE (Groups B and C) to those with HIVE Group D). Dots represent genes found to have differential expression in both studies. The x-axis represents log_2_ fold changes in frontal neocortex from Masliah et al. The y-axis represents log_2_ fold changes from frontal neocortex of the current study (groups B and C vs. group D). A highly significant correlation was observed overall (R = 0.73, *p* = 3.2^−17^). The strong correlation between the two studies was strongly driven by increased expression of several immune-related genes across both studies, shown in the right margin.
(TIF)

Figure S3 **Principal Components Analysis (PCA) of Group D (HIV positive with NCI and with HIVE).** The cases in Group D were subjected to additional analysis due to the subsequent identification of co-infection in the brain of one subject (D5) with Cytomegalovirus and Papovavirus. Examination of the top two principal components reveals that in all three regions of the brain, D5 (triangle) clusters similar to the majority of the other cases in this group.
(TIF)

Table S1 Posthybridization RNA quality of white matter (sample #1 through #24).
(XLS)

Table S2 Posthybridization RNA quality of frontal neocortex (sample #25 through #48).
(XLS)

Table S3 Posthybridization RNA quality of basal ganglia (sample #49 through #72).
(XLS)

Table S4 List of significantly regulated transcripts with at least a two-fold change using Ingenuity Systems Pathways Analysis. Comparisons are for groups A versus B, A versus C, and A versus D. Three different brain sectors are given: WM is frontal white matter; FC is frontal neocortex; BG is basal ganglia.
(XLS)

Table S5 Transcripts significantly regulated, with at least a two-fold change using Ingenuity Systems Pathway Analysis, in more than one brain region in subjects with neurocognitive impairment and HIVE (Group A vs D).
(XLS)

Table S6 List of transcripts significantly regulated with at least a two-fold change in Group C (HIV-associated neurocognitive impairment without HIV encephalitis) using Ingenuity Systems Pathway Analysis.
(XLS)

Table S7 List of transcripts significantly regulated with at least a two-fold change in Group B (HIV-infected subjects without neurocognitive impairment or encephalitis) using Ingenuity Systems Pathway Analysis.
(XLS)

Table S8 List of correlation coefficients between brain gene expression and brain HIV RNA concentration.
(XLSX)

Table S9 Correlation coefficients between brain gene expression and brain HIV RNA concentration in three separate brain sectors.
(XLSX)

## References

[1] Budka H (1991) Neuropathology of human immunodeficiency virus infection. Brain Pathol 1: 163–175. 1669705 10.1111/j.1750-3639.1991.tb00656.x

[2] Glass JD, Fedor H, Wesselingh SL, McArthur JC (1995) Immunocytochemical quantitation of human immunodeficiency virus in the brain: correlations with dementia. Ann Neurol 38: 755–762. 7486867 10.1002/ana.410380510

[3] Glass JD, Wesselingh SL, Selnes OA, McArthur JC (1993) Clinical-neuropathologic correlation in HIV-associated dementia. Neurology 43: 2230–2237. 8232935 10.1212/wnl.43.11.2230

[4] Wiley CA, Achim C (1994) Human immunodeficiency virus encephalitis is the pathological correlate of dementia in acquired immunodeficiency syndrome. Ann Neurol 36: 673–676. 7944304 10.1002/ana.410360422

[5] Everall I, Vaida F, Khanlou N, Lazzaretto D, Achim C, et al (2009) Cliniconeuropathologic correlates of human immunodeficiency virus in the era of antiretroviral therapy. J Neurovirol 15: 360–370. 20175693 10.3109/13550280903131915 PMC3078805

[6] Masliah E, DeTeresa RM, Mallory ME, Hansen LA (2000) Changes in pathological findings at autopsy in AIDS cases for the last 15 years. AIDS 14: 69–74. 10714569 10.1097/00002030-200001070-00008

[7] Morgello S, Mahboob R, Yakoushina T, Khan S, Hague K (2002) Autopsy findings in a human immunodeficiency virus-infected population over 2 decades: influences of gender, ethnicity, risk factors, and time. Arch Pathol Lab Med 126: 182–190. 11825115 10.5858/2002-126-0182-AFIAHI

[8] Heaton RK, Clifford DB, Franklin DR Jr, Woods SP, Ake C, et al (2010) HIV-associated neurocognitive disorders persist in the era of potent antiretroviral therapy: CHARTER Study. Neurology 75: 2087–2096. 21135382 10.1212/WNL.0b013e318200d727 PMC2995535

[9] Heaton RK, Grant I, Butters N, White DA, Kirson D, et al (1995) The HNRC 500–neuropsychology of HIV infection at different disease stages. HIV Neurobehavioral Research Center. J Int Neuropsychol Soc 1: 231–251. 9375218 10.1017/s1355617700000230

[10] McArthur JC (2004) HIV dementia: an evolving disease. J Neuroimmunol 157: 3–10. 15579274 10.1016/j.jneuroim.2004.08.042

[11] Antinori A, Arendt G, Becker JT, Brew BJ, Byrd DA, et al (2007) Updated research nosology for HIV-associated neurocognitive disorders. Neurology 69: 1789–1799. 17914061 10.1212/01.WNL.0000287431.88658.8b PMC4472366

[12] Everall I, Salaria S, Roberts E, Corbeil J, Sasik R, et al (2005) Methamphetamine stimulates interferon inducible genes in HIV infected brain. J Neuroimmunol 170: 158–171. 16249037 10.1016/j.jneuroim.2005.09.009

[13] Gelman BB, Spencer JA, Holzer CE 3rd, Soukup VM (2006) Abnormal striatal dopaminergic synapses in National NeuroAIDS Tissue Consortium subjects with HIV encephalitis. J Neuroimmune Pharmacol 1: 410–420. 18040813 10.1007/s11481-006-9030-6

[14] Masliah E, Roberts ES, Langford D, Everall I, Crews L, et al (2004) Patterns of gene dysregulation in the frontal cortex of patients with HIV encephalitis. J Neuroimmunol 157: 163–175. 15579294 10.1016/j.jneuroim.2004.08.026

[15] Roberts ES, Masliah E, Fox HS (2004) CD163 identifies a unique population of ramified microglia in HIV encephalitis (HIVE). J Neuropathol Exp Neurol 63: 1255–1264. 15624762 10.1093/jnen/63.12.1255

[16] Roberts ES, Zandonatti MA, Watry DD, Madden LJ, Henriksen SJ, et al (2003) Induction of pathogenic sets of genes in macrophages and neurons in NeuroAIDS. Am J Pathol 162: 2041–2057. 12759259 10.1016/S0002-9440(10)64336-2 PMC1868118

[17] Burdo TH, Ellis RJ, Fox HS (2008) Osteopontin is increased in HIV-associated dementia. J Infect Dis 198: 715–722. 18616394 10.1086/590504 PMC2587027

[18] Gelman BB, Soukup VM, Schuenke KW, Keherly MJ, Holzer C 3rd, et al (2004) Acquired neuronal channelopathies in HIV-associated dementia. J Neuroimmunol 157: 111–119. 15579287 10.1016/j.jneuroim.2004.08.044

[19] Alirezaei M, Kiosses WB, Flynn CT, Brady NR, Fox HS (2008) Disruption of neuronal autophagy by infected microglia results in neurodegeneration. PLoS One 3: e2906. 18682838 10.1371/journal.pone.0002906 PMC2483417

[20] Gelman BB, Soukup VM, Holzer CE, 3rd, Fabian RH, Schuenke KW, et al (2005) Potential role for white matter lysosome expansion in HIV-associated dementia. J Acquir Immune Defic Syndr 39: 422–425. 16010164 10.1097/01.qai.0000164250.41475.f2

[21] Morgello S, Gelman BB, Kozlowski PB, Vinters HV, Masliah E, et al (2001) The National NeuroAIDS Tissue Consortium: a new paradigm in brain banking with an emphasis on infectious disease. Neuropathol Appl Neurobiol 27: 326–335. 11532163 10.1046/j.0305-1846.2001.00334.x

[22] Jernigan TL, Archibald SL, Fennema-Notestine C, Taylor MJ, Theilmann RJ, et al (2011) Clinical factors related to brain structure in HIV: the CHARTER study. J Neurovirol 17: 248–257. 21544705 10.1007/s13365-011-0032-7 PMC3702821

[23] Langford TD, Letendre SL, Marcotte TD, Ellis RJ, McCutchan JA, et al (2002) Severe, demyelinating leukoencephalopathy in AIDS patients on antiretroviral therapy. AIDS 16: 1019–1029. 11953468 10.1097/00002030-200205030-00008 PMC3548569

[24] Paul RH, Yiannoutsos CT, Miller EN, Chang L, Marra CM, et al (2007) Proton MRS and neuropsychological correlates in AIDS dementia complex: evidence of subcortical specificity. J Neuropsychiatry Clin Neurosci 19: 283–292. 17827413 10.1176/jnp.2007.19.3.283

[25] Schmidbauer M, Huemer M, Cristina S, Trabattoni GR, Budka H (1992) Morphological spectrum, distribution and clinical correlation of white matter lesions in AIDS brains. Neuropathol Appl Neurobiol 18: 489–501. 1454139 10.1111/j.1365-2990.1992.tb00816.x

[26] Xing HQ, Moritoyo T, Mori K, Tadakuma K, Sugimoto C, et al (2003) Simian immunodeficiency virus encephalitis in the white matter and degeneration of the cerebral cortex occur independently in simian immunodeficiency virus-infected monkey. J Neurovirol 9: 508–518. 12907395 10.1080/13550280390218904

[27] Achim CL, Wang R, Miners DK, Wiley CA (1994) Brain viral burden in HIV infection. J Neuropathol Exp Neurol 53: 284–294. 8176412 10.1097/00005072-199405000-00010

[28] Buesa C, Maes T, Subirada F, Barrachina M, Ferrer I (2004) DNA chip technology in brain banks: confronting a degrading world. J Neuropathol Exp Neurol 63: 1003–1014. 15535128 10.1093/jnen/63.10.1003

[29] Ervin JF, Heinzen EL, Cronin KD, Goldstein D, Szymanski MH, et al (2007) Postmortem delay has minimal effect on brain RNA integrity. J Neuropathol Exp Neurol 66: 1093–1099. 18090918 10.1097/nen.0b013e31815c196a

[30] Lee J, Hever A, Willhite D, Zlotnik A, Hevezi P (2005) Effects of RNA degradation on gene expression analysis of human postmortem tissues. FASEB J 19: 1356–1358. 15955843 10.1096/fj.04-3552fje

[31] Boehm U, Klamp T, Groot M, Howard JC (1997) Cellular responses to interferon-gamma. Annu Rev Immunol 15: 749–795. 9143706 10.1146/annurev.immunol.15.1.749

[32] Brooimans RA, Hiemstra PS, van der Ark AA, Sim RB, van Es LA, et al (1989) Biosynthesis of complement factor H by human umbilical vein endothelial cells. Regulation by T cell growth factor and IFN-gamma. J Immunol 142: 2024–2030. 2522130

[33] Furman C, Sieminski AL, Kwiatkowski AV, Rubinson DA, Vasile E, et al (2007) Ena/VASP is required for endothelial barrier function in vivo. J Cell Biol 179: 761–775. 17998398 10.1083/jcb.200705002 PMC2080895

[34] Jahroudi N, Lynch DC (1994) Endothelial-cell-specific regulation of von Willebrand factor gene expression. Mol Cell Biol 14: 999–1008. 7507210 10.1128/mcb.14.2.999-1008.1994 PMC358455

[35] Jefferies WA, Brandon MR, Hunt SV, Williams AF, Gatter KC, et al (1984) Transferrin receptor on endothelium of brain capillaries. Nature 312: 162–163. 6095085 10.1038/312162a0

[36] McCarthy MJ, Crowther M, Bell PR, Brindle NP (1998) The endothelial receptor tyrosine kinase tie-1 is upregulated by hypoxia and vascular endothelial growth factor. FEBS Lett 423: 334–338. 9515734 10.1016/s0014-5793(98)00122-7

[37] Ohno M, Cooke JP, Dzau VJ, Gibbons GH (1995) Fluid shear stress induces endothelial transforming growth factor beta-1 transcription and production. Modulation by potassium channel blockade. J Clin Invest 95: 1363–1369. 7883983 10.1172/JCI117787 PMC441476

[38] Ripoche J, Mitchell JA, Erdei A, Madin C, Moffatt B, et al (1988) Interferon gamma induces synthesis of complement alternative pathway proteins by human endothelial cells in culture. J Exp Med 168: 1917–1922. 2972796 10.1084/jem.168.5.1917 PMC2189095

[39] Tar K, Birukova AA, Csortos C, Bako E, Garcia JG, et al (2004) Phosphatase 2A is involved in endothelial cell microtubule remodeling and barrier regulation. J Cell Biochem 92: 534–546. 15156565 10.1002/jcb.20036

[40] Vastag M, Skopal J, Kramer J, Kolev K, Voko Z, et al (1998) Endothelial cells cultured from human brain microvessels produce complement proteins factor H, factor B, C1 inhibitor, and C4. Immunobiology 199: 5–13. 9717663 10.1016/S0171-2985(98)80059-4

[41] Oide T, Yoshida K, Kaneko K, Ohta M, Arima K (2006) Iron overload and antioxidative role of perivascular astrocytes in aceruloplasminemia. Neuropathol Appl Neurobiol 32: 170–176. 16599945 10.1111/j.1365-2990.2006.00710.x

[42] de Chaldee M, Gaillard MC, Bizat N, Buhler JM, Manzoni O, et al (2003) Quantitative assessment of transcriptome differences between brain territories. Genome Res 13: 1646–1653. 12840043 10.1101/gr.1173403 PMC403738

[43] Greengard P (2001) The neurobiology of slow synaptic transmission. Science 294: 1024–1030. 11691979 10.1126/science.294.5544.1024

[44] Deutch AY, Colbran RJ, Winder DJ (2007) Striatal plasticity and medium spiny neuron dendritic remodeling in parkinsonism. Parkinsonism Relat Disord 13 Suppl 3S251–258. 18267246 10.1016/S1353-8020(08)70012-9 PMC4820336

[45] Surmeier DJ, Ding J, Day M, Wang Z, Shen W (2007) D1 and D2 dopamine-receptor modulation of striatal glutamatergic signaling in striatal medium spiny neurons. Trends Neurosci 30: 228–235. 17408758 10.1016/j.tins.2007.03.008

[46] Lane HC, Masur H, Edgar LC, Whalen G, Rook AH, et al (1983) Abnormalities of B-cell activation and immunoregulation in patients with the acquired immunodeficiency syndrome. N Engl J Med 309: 453–458. 6224088 10.1056/NEJM198308253090803

[47] Gelman BB (2007) The neuropathology of HIV. Handb Clin Neurol 85: 301–317. 18808989 10.1016/S0072-9752(07)85018-4

[48] Gray F, Chretien F, Vallat-Decouvelaere AV, Scaravilli F (2003) The changing pattern of HIV neuropathology in the HAART era. J Neuropathol Exp Neurol 62: 429–440. 12769183 10.1093/jnen/62.5.429

[49] Masliah E, Heaton RK, Marcotte TD, Ellis RJ, Wiley CA, et al (1997) Dendritic injury is a pathological substrate for human immunodeficiency virus-related cognitive disorders. HNRC Group. The HIV Neurobehavioral Research Center. Ann Neurol 42: 963–972. 9403489 10.1002/ana.410420618

[50] Fox HS (2008) Virus-host interaction in the simian immunodeficiency virus-infected brain. J Neurovirol 14: 286–291. 18780229 10.1080/13550280802132824 PMC2665180

[51] Roberts ES, Huitron-Resendiz S, Taffe MA, Marcondes MC, Flynn CT, et al (2006) Host response and dysfunction in the CNS during chronic simian immunodeficiency virus infection. J Neurosci 26: 4577–4585. 16641237 10.1523/JNEUROSCI.4504-05.2006 PMC6674066

[52] Kaul M, Garden GA, Lipton SA (2001) Pathways to neuronal injury and apoptosis in HIV-associated dementia. Nature 410: 988–994. 11309629 10.1038/35073667

[53] Williams KC, Hickey WF (2002) Central nervous system damage, monocytes and macrophages, and neurological disorders in AIDS. Annu Rev Neurosci 25: 537–562. 12052920 10.1146/annurev.neuro.25.112701.142822

[54] Potash MJ, Chao W, Bentsman G, Paris N, Saini M, et al (2005) A mouse model for study of systemic HIV-1 infection, antiviral immune responses, and neuroinvasiveness. Proc Natl Acad Sci U S A 102: 3760–3765. 15728729 10.1073/pnas.0500649102 PMC553332

[55] Roberts ES, Burudi EM, Flynn C, Madden LJ, Roinick KL, et al (2004) Acute SIV infection of the brain leads to upregulation of IL6 and interferon-regulated genes: expression patterns throughout disease progression and impact on neuroAIDS. J Neuroimmunol 157: 81–92. 15579284 10.1016/j.jneuroim.2004.08.030

[56] Sas AR, Bimonte-Nelson HA, Tyor WR (2007) Cognitive dysfunction in HIV encephalitic SCID mice correlates with levels of Interferon-alpha in the brain. AIDS 21: 2151–2159. 18090041 10.1097/QAD.0b013e3282f08c2f

[57] Zink MC, Brice AK, Kelly KM, Queen SE, Gama L, et al (2010) Simian immunodeficiency virus-infected macaques treated with highly active antiretroviral therapy have reduced central nervous system viral replication and inflammation but persistence of viral DNA. J Infect Dis 202: 161–170. 20497048 10.1086/653213 PMC2880623

[58] Masliah E, Ge N, Achim CL, Wiley CA (1995) Differential vulnerability of calbindin-immunoreactive neurons in HIV encephalitis. J Neuropathol Exp Neurol 54: 350–357. 7745434 10.1097/00005072-199505000-00008

[59] Tepper JM, Koos T, Wilson CJ (2004) GABAergic microcircuits in the neostriatum. Trends Neurosci 27: 662–669. 15474166 10.1016/j.tins.2004.08.007

[60] Benes FM, Berretta S (2001) GABAergic interneurons: implications for understanding schizophrenia and bipolar disorder. Neuropsychopharmacology 25: 1–27. 11377916 10.1016/S0893-133X(01)00225-1

[61] Hashimoto T, Bazmi HH, Mirnics K, Wu Q, Sampson AR, et al (2008) Conserved regional patterns of GABA-related transcript expression in the neocortex of subjects with schizophrenia. Am J Psychiatry 165: 479–489. 18281411 10.1176/appi.ajp.2007.07081223 PMC2894608

[62] Markram H, Toledo-Rodriguez M, Wang Y, Gupta A, Silberberg G, et al (2004) Interneurons of the neocortical inhibitory system. Nat Rev Neurosci 5: 793–807. 15378039 10.1038/nrn1519

[63] Sequeira A, Mamdani F, Ernst C, Vawter MP, Bunney WE, et al (2009) Global brain gene expression analysis links glutamatergic and GABAergic alterations to suicide and major depression. PLoS One 4: e6585. 19668376 10.1371/journal.pone.0006585 PMC2719799

[64] Sibille E, Morris HM, Kota RS, Lewis DA (2011) GABA-related transcripts in the dorsolateral prefrontal cortex in mood disorders. Int J Neuropsychopharmacol 14: 721–734. 21226980 10.1017/S1461145710001616 PMC3388740

[65] Ke Y, Streeter CC, Nassar LE, Sarid-Segal O, Hennen J, et al (2004) Frontal lobe GABA levels in cocaine dependence: a two-dimensional, J-resolved magnetic resonance spectroscopy study. Psychiatry Res 130: 283–293. 15135161 10.1016/j.pscychresns.2003.12.001

[66] Baldeweg T, Gruzelier JH (1997) Alpha EEG activity and subcortical pathology in HIV infection. Int J Psychophysiol 26: 431–442. 9203019 10.1016/s0167-8760(97)00780-0

[67] Masliah E, Ge N, Achim CL, Hansen LA, Wiley CA (1992) Selective neuronal vulnerability in HIV encephalitis. J Neuropathol Exp Neurol 51: 585–593. 1484289 10.1097/00005072-199211000-00003

[68] An SF, Giometto B, Groves M, Miller RF, Beckett AA, et al (1997) Axonal damage revealed by accumulation of beta-APP in HIV-positive individuals without AIDS. J Neuropathol Exp Neurol 56: 1262–1268. 9370237 10.1097/00005072-199711000-00011

[69] Gelman BB, Nguyen TP (2010) Synaptic proteins linked to HIV-1 infection and immunoproteasome induction: proteomic analysis of human synaptosomes. J Neuroimmune Pharmacol 5: 92–102. 19693676 10.1007/s11481-009-9168-0 PMC2824116

[70] Banks WA (2006) The blood-brain barrier as a regulatory interface in the gut-brain axes. Physiol Behav 89: 472–476. 16904139 10.1016/j.physbeh.2006.07.004

[71] Hawkins BT, Davis TP (2005) The blood-brain barrier/neurovascular unit in health and disease. Pharmacol Rev 57: 173–185. 15914466 10.1124/pr.57.2.4

[72] de Larranaga GF, Bocassi AR, Puga LM, Alonso BS, Benetucci JA (2003) Endothelial markers and HIV infection in the era of highly active antiretroviral treatment. Thromb Res 110: 93–98. 12893023 10.1016/s0049-3848(03)00291-3

[73] Kristoffersen US, Kofoed K, Kronborg G, Giger AK, Kjaer A, et al (2009) Reduction in circulating markers of endothelial dysfunction in HIV-infected patients during antiretroviral therapy. HIV Med 10: 79–87. 19200170 10.1111/j.1468-1293.2008.00661.x

[74] Melendez MM, McNurlan MA, Mynarcik DC, Khan S, Gelato MC (2008) Endothelial adhesion molecules are associated with inflammation in subjects with HIV disease. Clin Infect Dis 46: 775–780. 18225982 10.1086/527563

[75] Ross AC, Armentrout R, O’Riordan MA, Storer N, Rizk N, et al (2008) Endothelial activation markers are linked to HIV status and are independent of antiretroviral therapy and lipoatrophy. J Acquir Immune Defic Syndr 49: 499–506. 18989230 10.1097/QAI.0b013e318189a794 PMC2778267

[76] Abbott NJ, Ronnback L, Hansson E (2006) Astrocyte-endothelial interactions at the blood-brain barrier. Nat Rev Neurosci 7: 41–53. 16371949 10.1038/nrn1824

[77] Banks WA, Ercal N, Price TO (2006) The blood-brain barrier in neuroAIDS. Curr HIV Res 4: 259–266. 16842079 10.2174/157016206777709447

[78] Power C, Kong PA, Crawford TO, Wesselingh S, Glass JD, et al (1993) Cerebral white matter changes in acquired immunodeficiency syndrome dementia: alterations of the blood-brain barrier. Ann Neurol 34: 339–350. 7689819 10.1002/ana.410340307

[79] Toborek M, Lee YW, Flora G, Pu H, Andras IE, et al (2005) Mechanisms of the blood-brain barrier disruption in HIV-1 infection. Cell Mol Neurobiol 25: 181–199. 15962513 10.1007/s10571-004-1383-x PMC11529531

[80] Ancuta P, Kamat A, Kunstman KJ, Kim EY, Autissier P, et al (2008) Microbial translocation is associated with increased monocyte activation and dementia in AIDS patients. PLoS One 3: e2516. 18575590 10.1371/journal.pone.0002516 PMC2424175

[81] Aird WC (2003) The role of the endothelium in severe sepsis and multiple organ dysfunction syndrome. Blood 101: 3765–3777. 12543869 10.1182/blood-2002-06-1887

[82] Wang Q, Du F, Qian ZM, Ge XH, Zhu L, et al (2008) Lipopolysaccharide induces a significant increase in expression of iron regulatory hormone hepcidin in the cortex and substantia nigra in rat brain. Endocrinology 149: 3920–3925. 18450970 10.1210/en.2007-1626 PMC2488231

[83] Zhao B, Bowden RA, Stavchansky SA, Bowman PD (2001) Human endothelial cell response to gram-negative lipopolysaccharide assessed with cDNA microarrays. Am J Physiol Cell Physiol 281: C1587–1595. 11600422 10.1152/ajpcell.2001.281.5.C1587

[84] Nath A, Anderson C, Jones M, Maragos W, Booze R, et al (2000) Neurotoxicity and dysfunction of dopaminergic systems associated with AIDS dementia. J Psychopharmacol 14: 222–227. 11106300 10.1177/026988110001400305

[85] Wang GJ, Chang L, Volkow ND, Telang F, Logan J, et al (2004) Decreased brain dopaminergic transporters in HIV-associated dementia patients. Brain 127: 2452–2458. 15319273 10.1093/brain/awh269

[86] Ernst T, Chang L, Jovicich J, Ames N, Arnold S (2002) Abnormal brain activation on functional MRI in cognitively asymptomatic HIV patients. Neurology 59: 1343–1349. 12427881 10.1212/01.wnl.0000031811.45569.b0

[87] Melrose RJ, Tinaz S, Castelo JM, Courtney MG, Stern CE (2008) Compromised fronto-striatal functioning in HIV: an fMRI investigation of semantic event sequencing. Behav Brain Res 188: 337–347. 18242723 10.1016/j.bbr.2007.11.021

[88] Borjabad A, Morgello S, Chao W, Kim SY, Brooks AI, et al (2011) Significant effects of antiretroviral therapy on global gene expression in brain tissues of patients with HIV-1-associated neurocognitive disorders. PLoS Pathog 7: e1002213. 21909266 10.1371/journal.ppat.1002213 PMC3164642

[89] Woods SP, Rippeth JD, Frol AB, Levy JK, Ryan E, et al (2004) Interrater reliability of clinical ratings and neurocognitive diagnoses in HIV. J Clin Exp Neuropsychol 26: 759–778. 15370374 10.1080/13803390490509565

[90] Schroeder A, Mueller O, Stocker S, Salowsky R, Leiber M, et al (2006) The RIN: an RNA integrity number for assigning integrity values to RNA measurements. BMC Mol Biol 7: 3. 16448564 10.1186/1471-2199-7-3 PMC1413964

[91] Palmer S, Wiegand AP, Maldarelli F, Bazmi H, Mican JM, et al (2003) New real-time reverse transcriptase-initiated PCR assay with single-copy sensitivity for human immunodeficiency virus type 1 RNA in plasma. J Clin Microbiol 41: 4531–4536. 14532178 10.1128/JCM.41.10.4531-4536.2003 PMC254331

[92] Boulesteix AL, Strimmer K (2007) Partial least squares: a versatile tool for the analysis of high-dimensional genomic data. Brief Bioinform 8: 32–44. 16772269 10.1093/bib/bbl016

[93] Jain N, Thatte J, Braciale T, Ley K, O’Connell M, et al (2003) Local-pooled-error test for identifying differentially expressed genes with a small number of replicated microarrays. Bioinformatics 19: 1945–1951. 14555628 10.1093/bioinformatics/btg264

[94] Benjamini Y, Drai D, Elmer G, Kafkafi N, Golani I (2001) Controlling the false discovery rate in behavior genetics research. Behav Brain Res 125: 279–284. 11682119 10.1016/s0166-4328(01)00297-2

[95] Calvano SE, Xiao W, Richards DR, Felciano RM, Baker HV, et al (2005) A network-based analysis of systemic inflammation in humans. Nature 437: 1032–1037. 16136080 10.1038/nature03985

