# Supplementary figures and images for: The National NeuroAIDS Tissue Consortium Brain Gene Array: Two Types of HIV-Associated Neurocognitive Impairment
Source: PLoS One. 2012 Sep 26;7(9):e46178. doi: 10.1371/journal.pone.0046178 (PMC3458860; doi:10.1371/journal.pone.0046178)

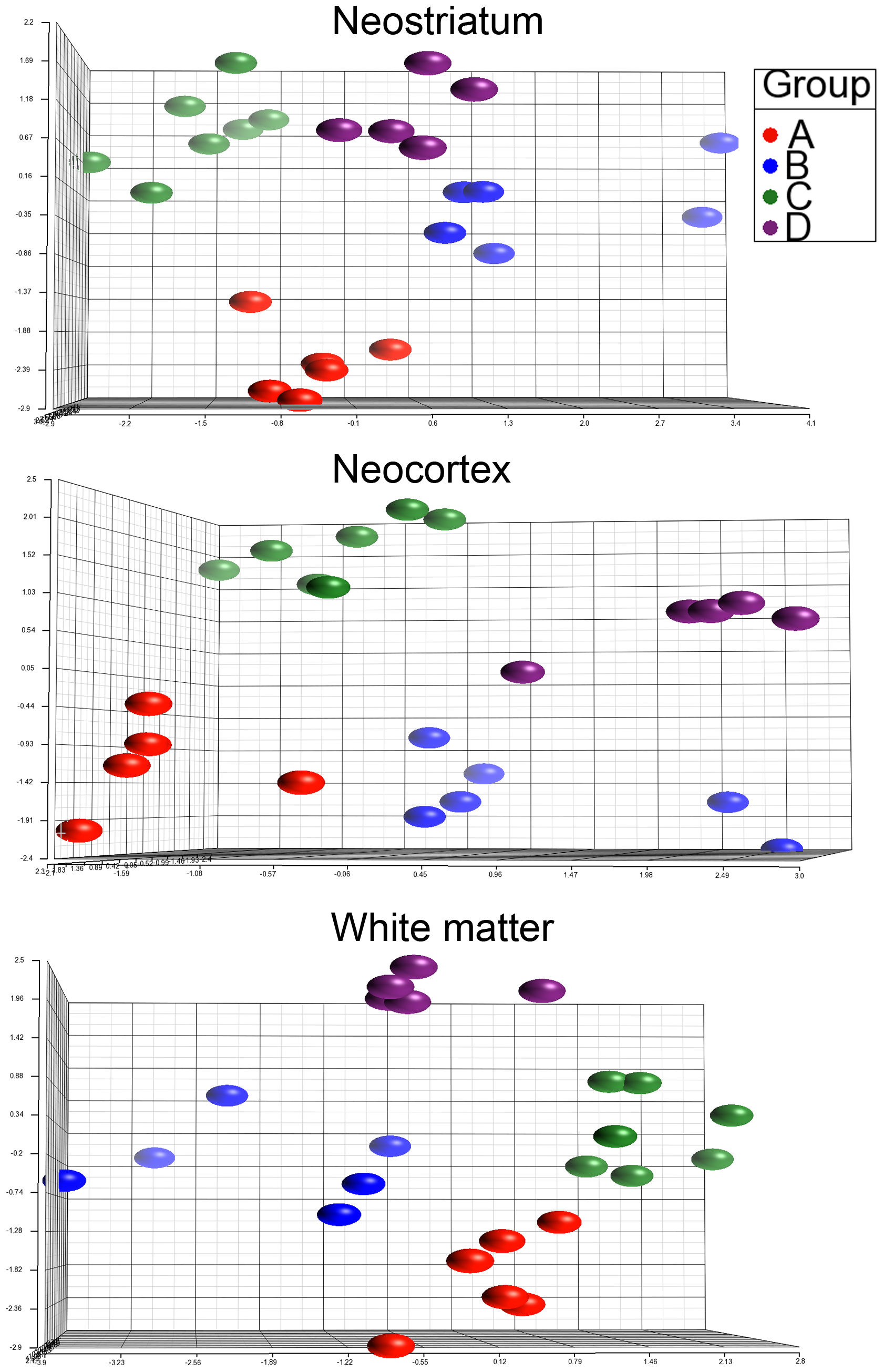

Supplement: Figure S1 — Partial least squares regression analysis. Global gene expression in the four groups (A: HIV negative; B: HIV positive without NCI or HIVE, C: HIV positive with NCI without HIVE, D; HIV positive with NCI and with HIVE) was analyzed within each brain region. Graphs show the distribution of samples in respect to the top three principal components. (TIF) [file pone.0046178.s001.tif]

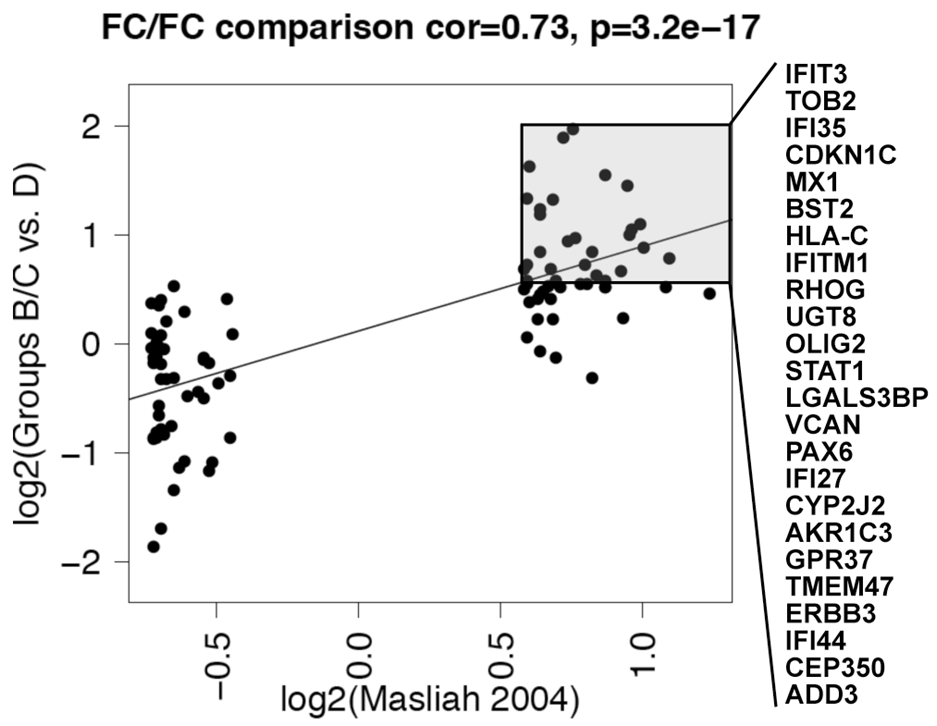

Supplement: Figure S2 — Neocortical gene expression in this study compared to prior array results. Expression in the array is similar to the array of Masliah et al (reference 14). Both studies compared HIV infected brain specimens without HIVE (Groups B and C) to those with HIVE Group D). Dots represent genes found to have differential expression in both studies. The x-axis represents log2 fold changes in frontal neocortex from Masliah et al. The y-axis represents log2 fold changes from frontal neocortex of the current study (groups B and C vs. group D). A highly significant correlation was observed overall (R = 0.73, p = 3.2−17). The strong correlation between the two studies was strongly driven by increased expression of several immune-related genes across both studies, shown in the right margin. (TIF) [file pone.0046178.s002.tif]

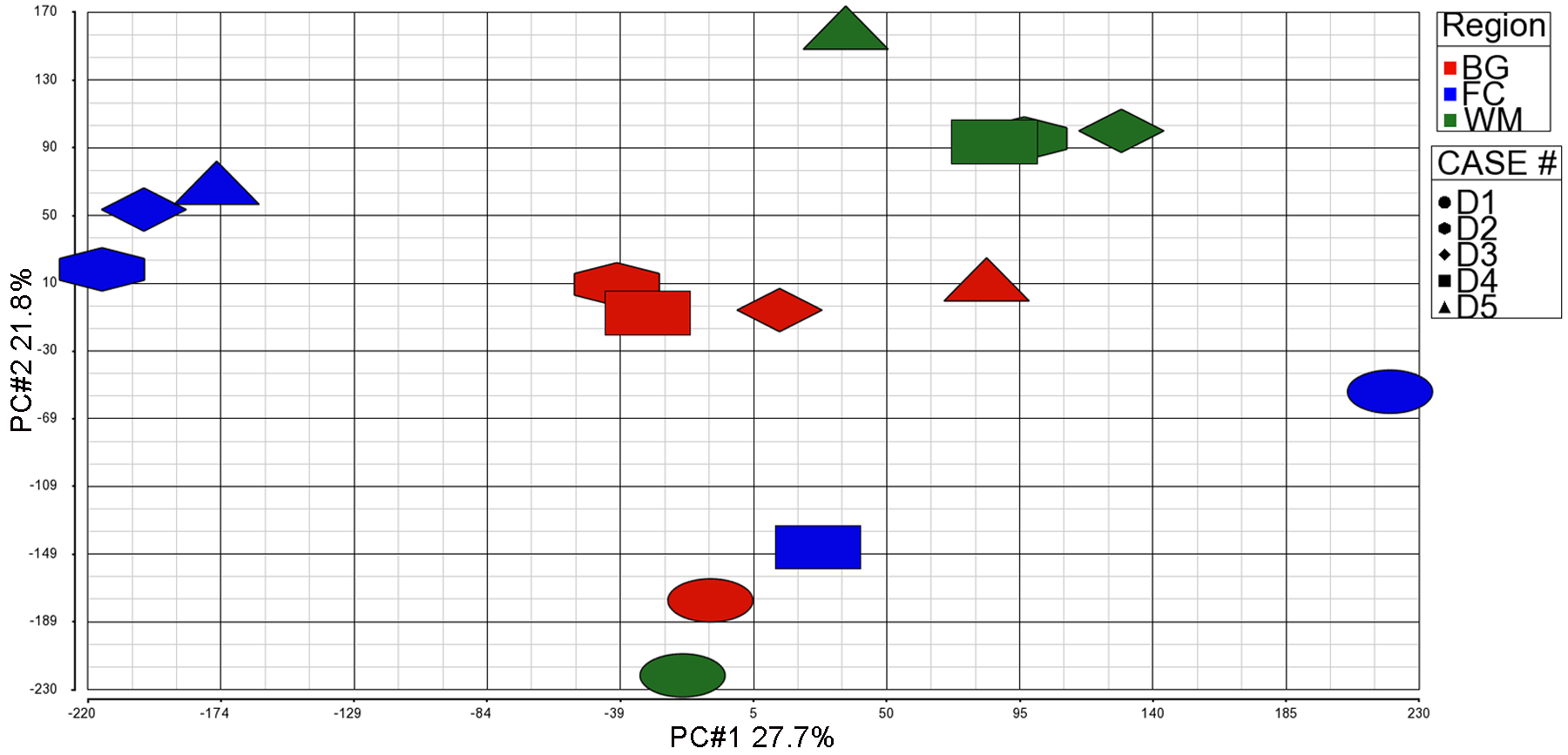

Supplement: Figure S3 — Principal Components Analysis (PCA) of Group D (HIV positive with NCI and with HIVE). The cases in Group D were subjected to additional analysis due to the subsequent identification of co-infection in the brain of one subject (D5) with Cytomegalovirus and Papovavirus. Examination of the top two principal components reveals that in all three regions of the brain, D5 (triangle) clusters similar to the majority of the other cases in this group. (TIF) [file pone.0046178.s003.tif]
